# Supplementary material for: Titanium Oxide Microspheres with Tunable Size and Phase Composition
Source: Materials (Basel). 2019 May 7;12(9):1472. doi: 10.3390/ma12091472 (PMC6539129; doi:10.3390/ma12091472)
Supplement: Supplementary file 1 [file materials-12-01472-s001.pdf]

materials

Article

Titanium Oxide Microspheres with Tunable Size and Phase Composition

Anton S. Poluboyarinov [1], Vitaly I. Chelpanov [2], Vasily A. Lebedev [3], Daniil A. Kozlov [1,4], Kristina M. Khazova [1], Dmitry S. Volkov [3], Irina V. Kolesnik [1,3,4] and Alexey A. Garshev [1,3,*]

1 Faculty of Materials Science, Lomonosov Moscow State University, Leninskiye Hills 1, Moscow 119234, Russia; anpolvk@gmail.com (A.S.P.); danilkozlove@gmail.com (D.A.K.); khazovakm@gmail.com (K.M.K.); kolesnik.iv@gmail.com (I.V.K.)
2 Baikov Institute of Metallurgy and Material Science RAS, Leninsky Avenue 49, Moscow 119334, Russia; vitaliy.chelpanov@gmail.com
3 Faculty of Chemistry, Lomonosov Moscow State University, Leninskiye Hills 1, Moscow 119234, Russia; vasya_lebedev@mail.ru (V.A.L.); dmsvolkov@gmail.com (D.S.V.)
4 Institute of General Inorganic Chemistry RAS, Leninsky Avenue 31, Moscow 119071, Russia
* Correspondence: garshev@inorg.chem.msu.ru; Tel.: +7-495-939-4259

Supplementary information

1. Characterisation: Details

1.1. DCS

Differential disc centrifuge DC24000 UHR (CPS Instruments, Inc.; USA) was used for size distribution measurements by differential centrifugal sedimentation (DCS). The analyzer measures particle size distributions using centrifugal sedimentation within an optically clear spinning disc that is filled with fluid. Sedimentation is stabilized by a density gradient within the fluid, and accuracy of the measured sizes is insured by the use of the standard sample with known particle size for calibration. The photometric system for turbidity signal registration consists of a 405 nm laser diode and a detector. The DCCS Operating Software (CPS Instruments, Inc.; USA) was used to control centrifuge parameters and to calculate particle size distribution after each measurement. Suspensions of TiO2 are unstable in water therefore a mixture of deionized water and ethanol (1:1 by volume) was used for preparation of all suspensions for size distribution measurements by DCS. Particle refractive index value was 1.8 according to the literature data [1]. Particle density (2.35 g/cm^3) was measured by helium pycnometer. Non-sphericity factor 1 (ideal sphere) was used for calculations following SEM data.

Water suspension of monodispersed polystyrene latex particles ("Polymer Latex", Russia) was used for calibration. The working suspension 20 mg/L in water-ethanol mixture was prepared from initial 10 mass.% commercial suspension. The following parameters of polystyrene latex particles were used for all calculations: particle density 1.05 g/cm^3, particle size (1.32 $\pm$ 0.07) μm according to the TEM measurements of the manufacturer. Refractive index (1.627 at 405 nm) was obtained after extrapolation of known data [2]. Deionized water (18.2 MOhm·cm, produced by Milli-Q Integral system, Merck Millipore), ethanol 96 % v/v (Ph. Eur., pharma grade, PanReac AppliChem), D(+)-sucrose (pharma grade, PanReac AppliChem) were used for preparation of density gradient fluid and sample suspensions.

A gradient made from sucrose and water-ethanol mixture (1:1 by volume) was used. For this 8.0 g of sucrose was dissolved in 100 mL of the water-ethanol mixture, thereby a solution with sucrose concentration of 8 mass.% was obtained. Then solutions with sucrose concentrations of 0.8; 1.6; 2.4; 3.2; 4.0; 4.8; 5.6; 6.4; 7.2 mass.% were prepared using 8 mass.% bulk solution and pure water-ethanol mixture. Fluid density of 8 mass.% sucrose solution (0.95 g/cm^3) was measured by DA-640 specific

gravity meter (Kyoto Electronics Manufacturing Co., Ltd., Japan). Viscosity (1.20 cps) of 8 mass.% sucrose solution) was measured by µVISC (RheoSense, USA). Refractive index of 8 mass.% sucrose solution (1.367) was measured by RA600 refractometer (Kyoto Electronics Manufacturing Co., Ltd., Japan).

The optimal velocity of rotor (4004 rpm) was chosen automatically to prevent too fast sedimentation and was used for all measurements. After preparation 1.0 mL aliquot of each solution (beginning from 8 mass.% to clean water-ethanol mixture) was injected by syringe into the disk centrifuge to create a density gradient within the fluid inside the spinning rotor. Waiting period of approximately 10 to 15 min was required for the created step gradient to become linear. After that 100 µL of the standard sample were injected by syringe for calibration. After the standard size distribution was measured, 100 µL of sample suspension were injected. The standard solution was measured every 3 sample injections. Every sample was analysed three times, results were averaged.

1.2. TEM

Spherical particles were deposited on the surface of the steel substrate. Then platinum safety layers were deposited to the particles by trimethyl(methylcyclopentadienyl)platinum(IV) decomposition firstly under electron beam, then under low-current gallium focused ion beam and high-current gallium focused ion beam irradiation. Lamellae were prepared according to CrossBeam 1540 EsB (Zeiss) software procedure using focused ion beam (FIB) for TEM-sample preparation. After that samples were fixed on the Omniprobe Lift-Out grids then thinned by the ion beam and polished with the use of low-energy Ga-ion beam (2-5 keV).

1.3. DLS

Particles were suspended in 1:1 ethanol-water solution. Suspensions were treated with ultrasound using Bandelin SONOPULS HD 3100 homogenizer for 5 min at 10% amplitude. For each sample 3 measurements were made and the results were averaged. In case of systematic decrease of particle size with measurement number results of the first measurement was used.

1.4. ICP-MS

An additional synthesis with volume of 100 mL was carried out. Obtained mixture was separated into several parts and different amount of NaOH or water was added to each one. In 24 hours each sample was filtered through CHROMAFIL Xtra PTFE 0.2 µm syringe filters and dissolved in distilled concentrated HNO3. After that samples were diluted nearly 1000 times with deionized water to reach Ti concentration of approximately 1 ppm. Standard Ti solution ("Eco-analitica", Russia) was used for preparations of calibration standards covering concentration range from 0.1 ppm to 10 ppm.

1.5. Measurement of SPF

To measure SPF, the samples were dispersed in water-in-oil emulsion to make 10 wt. % mixture. The emulsion was prepared by slowly adding of phase A to phase B under thorough stirring. The composition of the phases is provided in Table S1. Then 0.1 mL of the mixture was distributed on the surface of Vitro-Skin® substrate (a dose of a product was 2 µL/cm^2) and dried for 15 min before recording of UV-Vis spectra.

Table S1. Composition of water-in-oil emulsion which was used for the measurement of SPF of TiO_2 microspheres.

	Component	Final Concentration, wt. %
Phase A	DI water	65.3
	Guar gum (Sigma-Aldrich)	0.2
	NaCl (99.5%, Sigma-Aldrich)	1.0
	Citric acid (99.5%, Sigma-Aldrich)	0.5
	Glycerol (99.5%, Sigma-Aldrich)	3.0
Phase B	Glyceryl trioctanoate (90%, Sigma-Aldrich)	22.0
	1-Hexadecanol (99%, Sigma-Aldrich)	4.0
	Emulsifier (Neocare P3R, Gobiotics ingredients)	4.0

2. Comparison of Methods for Particle Size Measurement

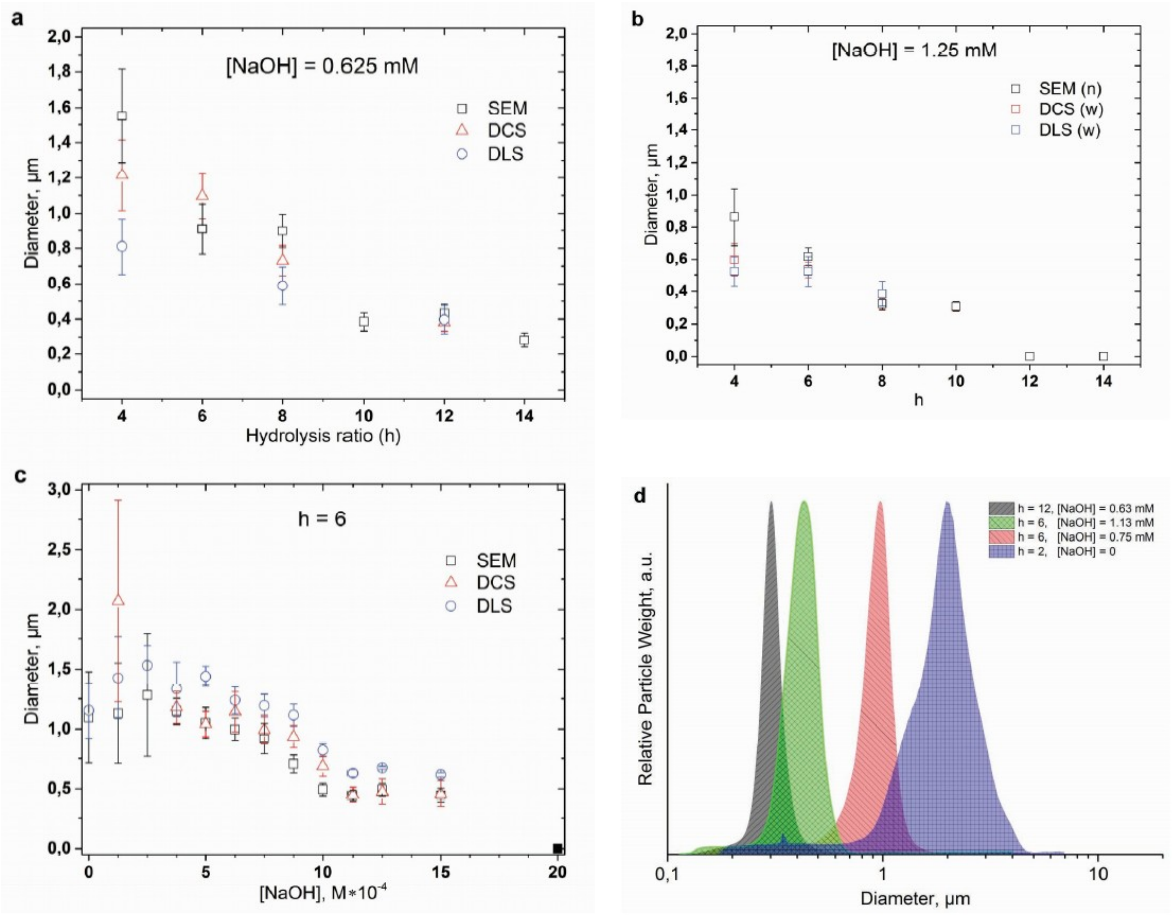

Figure S1. Comparison of methods for measuring particle size (SEM, DLS, DCS): mean diameter and its deviation of the samples synthesized with varying hydrolysis ratio at constant c(NaOH) = 0.625 mM (**a**) and 1.25 mM (**b**); with varying NaOH concentration at constant hydrolysis ratio h = 6 (**c**); particle size distributions obtained by DCS (**d**).

3. Aggregation Rate Constants and Stable Diameters

In order to illustrate it we calculated aggregation rate constants (B_{ij}) for titania particles at different surface potentials and ionic strengths. Calculations were performed basing on [3], Hamaker constant for titania was taken from [4], primary particle radius was set to 2 nm.

Particles are stabilized only when surface potential is high enough and ionic strength is low enough. We also calculated the stable particle size (the size at which aggregation rate constant is 1000

times lower than for primary particles at the same conditions [3]) at reasonable surface potentials and ionic strengths. Stable particle size can be viewed as a lower boundary of final particle size. It should be noted that lowest obtained stable particle diameter is about 200 nm which is close to experimentally obtained value of 250 nm.

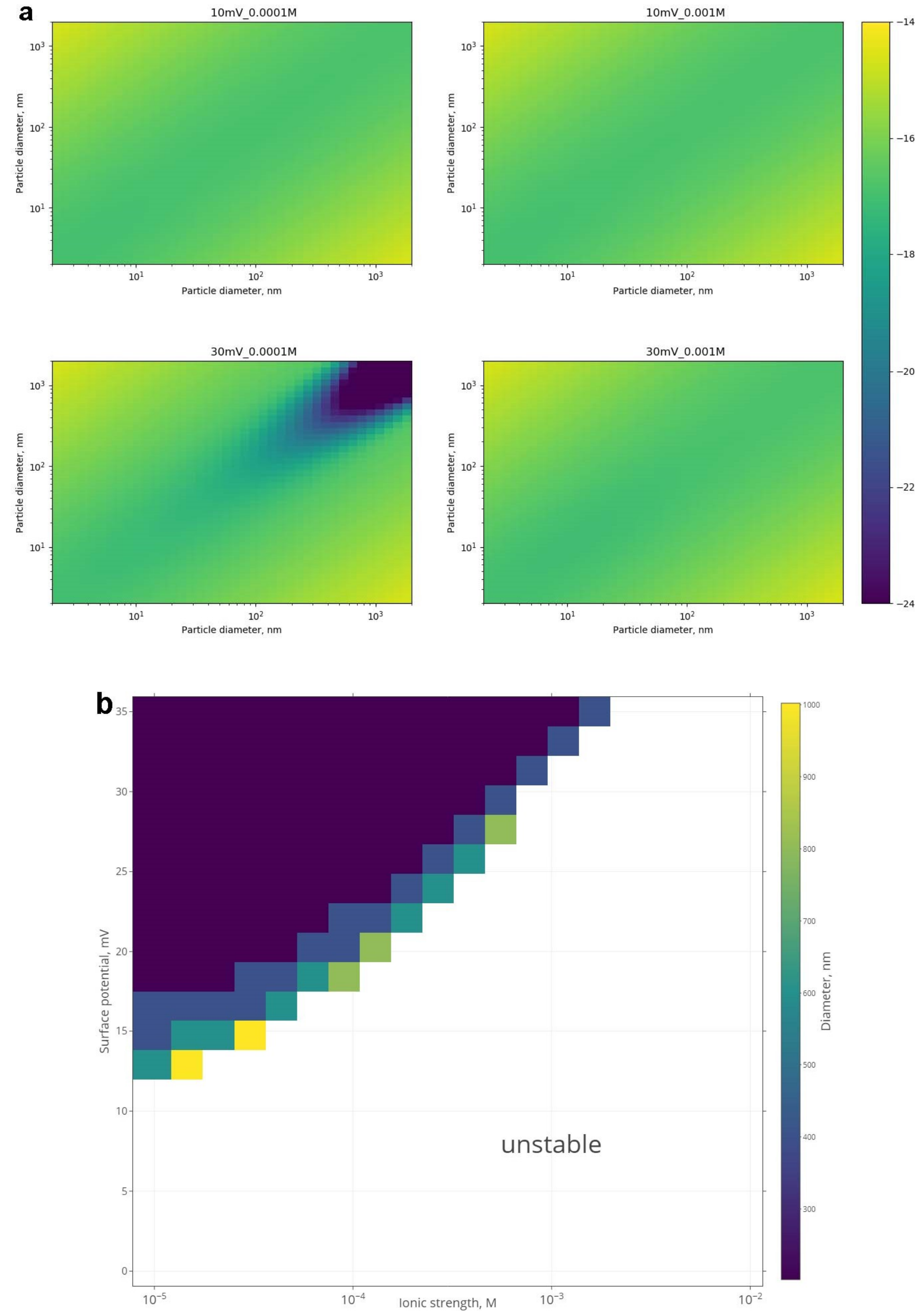

Figure S2. Aggregation rate constants (common logarithm) (**a**) and stable diameters (**b**) at different surface potentials and ionic strengths. Obtained values should be considered qualitative.

4. XRD, Nitrogen Sorption and Raman Spectra of the Annealed Samples

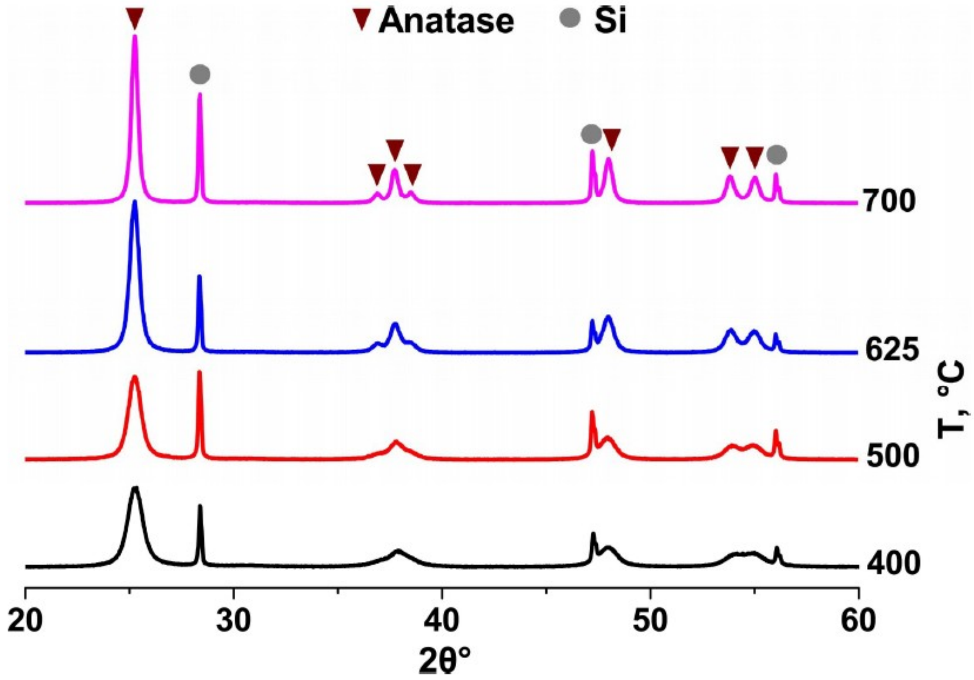

Figure S3. Powder XRD patterns of the annealed samples with Si as an internal standard. Samples annealed at temperatures in the range 400–700 °C consist of anatase in agreement with HTXRD data.

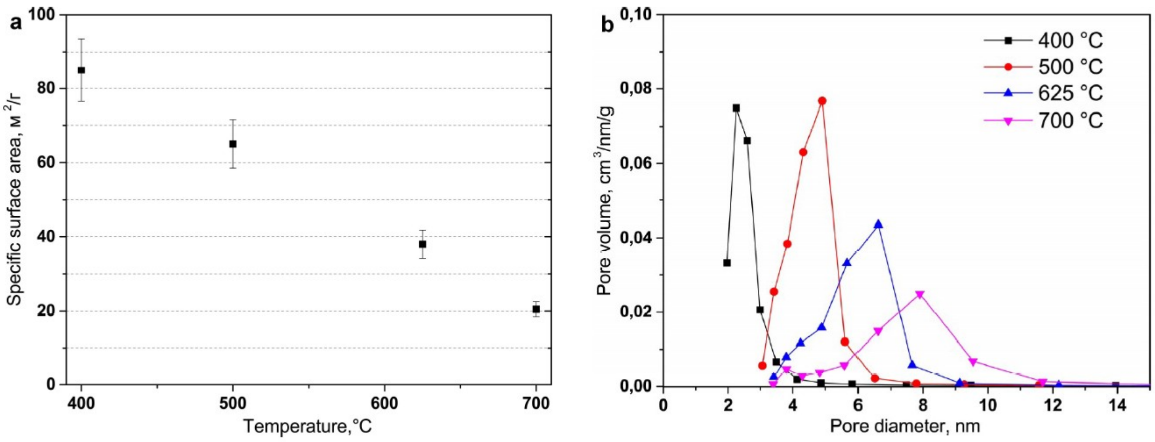

Figure S4. BET specific surface area (**a**) and BJH pore size distribution (**b**) of the annealed samples calculated using BET and BJH models respectively. Higher annealing temperatures correspond to lower SSA and higher pore size. Specific surface area decreases from 220 m^2/g to 20 m^2/g during annealing and mean pore size increases from 2.6 nm to 7.9 nm.

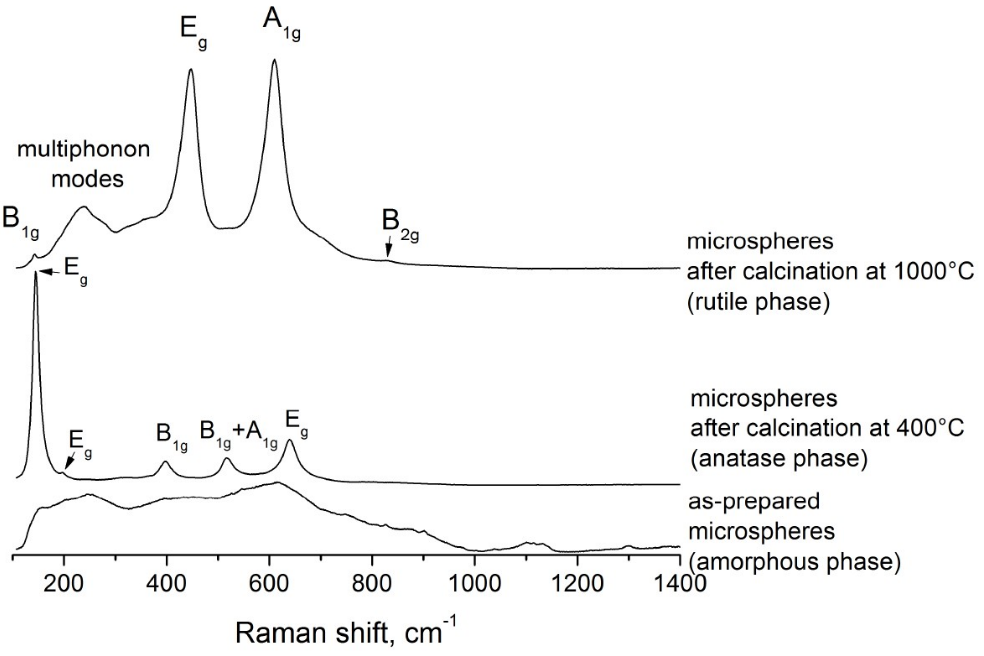

Figure 5. Raman spectra of the untreated, annealed at 400 °C and 1000 °C samples. Presence of the bands in the spectra of XRD-amorphous samples which are close to the bands of crystalline anatase and rutile indicates that the primary structure of amorphous phase comprises elements similar to crystalline phases.

References

1. Demirörs, A.F.; Jannasch, A.; Van Oostrum, P.D.J.; Schäffer, E.; Imhof, A.; Van Blaaderen, A. Seeded growth of titania colloids with refractive index tunability and fluorophore-free luminescence. *Langmuir* **2011**, *27*, 1626–1634.
2. Sultanova, N.; Kasarova, S.; Nikolov, I. Dispersion Properties of Optical Polymers. *Acta Phys. Pol. A* **2009**, *116*, 585–587.
3. Bogush, G.H.; Zukoski, C.F., IV. Uniform silica particle precipitation: An aggregative growth model. *J. Colloid Interface Sci.* **1991**, *142*, 19–34.
4. Bergstrm, L. Hamaker constants of inorganic materials. *J. Colloid Interface Sci.* **1997**, *70*, 125–169.
